# Analysis of the Chemical Profiles and Anti-*S. aureus* Activities of Essential Oils Extracted from Different Parts of Three Oregano Cultivars

**DOI:** 10.3390/foods10102328

**Published:** 2021-09-30

**Authors:** Yuanpeng Hao, Jingyi Li, Wenying Zhang, Meiyu Sun, Hui Li, Fei Xia, Hongxia Cui, Hongtong Bai, Lei Shi

**Affiliations:** 1 Key Laboratory of Plant Resources and Beijing Botanical Garden, Institute of Botany, Chinese Academy of Sciences, Beijing 100093, China; yphao@ibcas.ac.cn (Y.H.); jingyileesd@126.com (J.L.); wyzhang@ibcas.ac.cn (W.Z.); sunmeiyu@ibcas.ac.cn (M.S.); lihuibjfu@126.com (H.L.); 13651377226@163.com (F.X.); cuihongxia@ibcas.ac.cn (H.C.); bai13910086470@163.com (H.B.); 2 University of Chinese Academy of Sciences, Beijing 100049, China

**Keywords:** oregano essential oil, chemical composition, carvacrol, antibacterial activity, food safety

## Abstract

The use of antibiotics in the food industry is highly regulated owing to the potential harmful effects of antibiotics on human health. Therefore, it is crucial to seek alternatives for ensuring food safety. Essential oils (EOs) extracted from plants of the genus *Origanum* exhibit a wide range of chemical and antibacterial activities. Species and tissue factors shape the production and accumulation processes of EOs in *Origanum* plants, thereby affecting their bactericidal activity. In this study, the morphologies and EO yields from the inflorescences, leaves, and stems of three oregano cultivars were evaluated. In addition, the chemical compositions and antibacterial abilities of oregano EOs (OEOs) were assessed. The results showed that OEOs from the different parts of the plant displayed only minor differences in chemical composition, whereas the yield of EOs varied considerably. Additionally, the chemical profiles of OEOs differed significantly among cultivars. The carvacrol content in the OEOs was closely related to its activity against *Staphylococcus aureus*; the antibacterial properties of the OEOs were further verified using carvacrol. These findings suggested that OEOs possessing high antibacterial activity may have the potential to be developed as bactericides in the food industry.

## 1. Introduction

The genus *Origanum*, belonging to the family Labiatae, is a perennial herb native to the dry, rocky calcareous soils in mountainous areas; there are 39 species of *Origanum* worldwide, with wide distribution in the Mediterranean area [1]. Species within the genus are typically characterized based on morphological and chemical diversities. Oregano has been used as a food additive due to its antioxidant, antimicrobial, and antiparasitic activities [2].

According to estimates from the World Health Organization in 2015, 600 million people are affected every year by contaminated food, resulting in approximately 420,000 deaths annually [3]. *Staphylococcus aureus* is a Gram-positive foodborne pathogen that can easily contaminate high-protein foods, such as meat, eggs, and milk [4]. Prevention of contamination during food production, processing, and transport to consumers is an essential step for food safety and quality [5]. Currently, preventive measures and antibiotic treatments are the main approaches used to avoid food contamination. However, heavy use of antibiotics can lead to accumulation of drug resistance in bacteria [6]. Therefore, the selection of natural antibacterial agents is crucial for effective treatment of *S. aureus* infection [7]. 

Plants emit a large variety of volatile organic compounds (VOCs) during infection by pathogenic microbes. These compounds are often assumed to function in defense against pathogens [8]. Certain VOCs can be refined into essential oils (EOs) via steam distillation. EOs are commonly found in different parts of plants, including roots, stems, leaves, flowers, and fruits. Importantly, EOs are generally accepted by consumers due to their high volatility and ephemeral and biodegradable properties [9]. Dadalioglu and Evrendilek [10] reported that oregano EOs (OEOs) have strong antibacterial activity against common foodborne pathogens. From the perspective of reducing drug resistance, it is more informative to study the entire EO (complex mixtures of natural compounds) rather than several individual chemical components (single-molecule mode of action) due to their potential synergistic effect. These pronounced antioxidant and antibacterial capacities have made OEOs a promising replacement for antibiotics in the food industry.

The chemical characteristics and antibacterial activity of OEOs were reported in previous studies [2,6,9]. However, the OEO contents of various parts of different oregano cultivars have not been described in detail. Accordingly, in this study, we extracted and characterized OEOs from different parts of three oregano cultivars cultivated under the same experimental conditions. We then conducted a multidimensional exploration of OEO composition, and tested OEOs for antibacterial activity. Overall, our results provide important insights into the chemical characteristics and antibacterial properties of OEO extracts from different parts and oregano cultivars, supporting the use of oregano-derived compounds as food additives. 

## 2. Materials and Methods

### 2.1. Plant Materials, Bacterial Strains, and Standards

The oregano plants were grown in an experimental field of Institute of Botany, Chinese Academy of Sciences, in Nanyang, Henan Province, China (112°57 E, 32°78 N, altitude 116 m). The aerial parts were harvested at the full bloom stage and dried in the shade (20–25 °C) for further analyses. *S. aureus* CGMCC 1.4519 was obtained from the China General Microbiological Culture Collection Center and stored in Luria-Bertani (LB) broth with 25% glycerol (*v*/*v*) at −80 °C. Before each experiment, the test strain was cultured in LB broth for 18 h at 37 °C with shaking. Carvacrol was purchased from Aladdin Biochemical Technology Co., Ltd. (Shanghai, China).

### 2.2. Extraction of OEOs

Dried samples from different oregano parts were ground to a powder, and OEOs were extracted from 100 g powdered samples in the presence of distilled water (1000 mL) by steam distillation using a Clevenger apparatus. The process was carried out for 3 h, and the obtained OEO samples were dried over anhydrous sodium sulfate and stored in an amber bottle at 4 °C.

The EOs of different parts of *Origanum vulgare* ‘Hot & Spicy’ (Ovh), *O*. x *marjorana* ‘Hippokrates’ (Omh), and *O. vulgare* line 1 (Ovl) were designated Ovh inflorescence (Ovhi), Ovh leaf (Ovhl), Ovh stem (Ovhs), Omh inflorescence (Omhi), Omh leaf (Omhl), Omh stem (Omhs), Ovl inflorescence (Ovli), Ovl leaf (Ovll), and Ovl stem (Ovls), respectively. 

### 2.3. Gas Chromatography-Mass Spectrometry (GC-MS) Analysis of OEOs

The samples were then analyzed on a GC-MS system equipped with an HP-5MS column (Agilent Technologies, Santa Clara, CA, USA). The injector temperature was 250 °C. The oven program was conducted as follows: 1 μL of the sample was injected in a split mode of 40:1. The temperature was maintained at 40 °C for 2 min, the linear ramp reached 77 °C at a rate of 8 °C/min, and the second ramp reached 150 °C at a rate of 5 °C/min. The third ramp reached 185 °C at a rate of 3 °C/min. The fourth ramp reached 310 °C at a rate of 60 °C/min. The MS conditions were set as follows: electronic impact ion source temperature, 230 °C; ionization energy, 70 eV; quadrupole temperature, 150 °C; and mass range, 40–700 u. The EO compounds were identified by comparison of the NIST 17 mass spectral library with the retention index (RI) [11]. The RI values were determined using n-alkane hydrocarbons. The relative compound composition of the EOs was calculated based on the peak area.

### 2.4. Evaluation of Antibacterial Activity

#### 2.4.1. Diameter of the Inhibitory Zone (DIZ) Assays

The DIZ was measured using the disc diffusion method [12]. Briefly, 100 μL *S. aureus* suspensions (approximately 10^7^ CFU/mL) was evenly spread on the LB agar plates. Antimicrobial disks (6 mm in diameter) were placed on the surface of the test plates. Then, 6 μL of OEO was added to the disks. The plates were incubated at 37 °C for 24 h. The diameter of the zone of growth inhibition was measured using Vernier calipers (Airaj, Tsingtao, China). All experiments were performed in triplicate.

#### 2.4.2. Minimum Inhibitory Concentration (MIC) Assays

The OEOs were dissolved in LB broth and then serially diluted two-fold to achieve concentrations in the range 0.0625–128 mg/mL (0.0625, 0.125, 0.25, 0.5, 1, 2, 4, 8, 16, 32, 64, and 128 mg/mL). The MIC was measured using a 96-well microtiter plate dilution procedure. An inoculum suspension with a final concentration of approximately 10^7^ CFU/mL was added to a 96-well microplate; bacterial suspensions alone without OEO were inoculated as the control group. The MIC value was defined as the lowest concentration of OEOs where visible bacterial growth was not observed in the LB broth. The entire process required approximately 24 h at 37 °C.

### 2.5. Statistical Analysis

Data are expressed as means ± standard deviations. IBM SPSS statistics software (version 25.0) was used for statistical analysis. One-way analysis of variance was used to determine the significance of differences (*p* < 0.05). Heatmap analysis, principal component analysis (PCA) plots, UpSet plots, and Flower plots were evaluated using the R platform [13]. To further distinguish the differences and relationships of the three OEOs, a supervised statistical data treatment was performed using orthogonal projections to latent structures discriminant analysis (OPLS-DA) using SIMCA (Version 14.1; Umetrics, Umea, Sweden).

## 3. Results and Discussion

### 3.1. Morphological Observations

**Figure 1:**
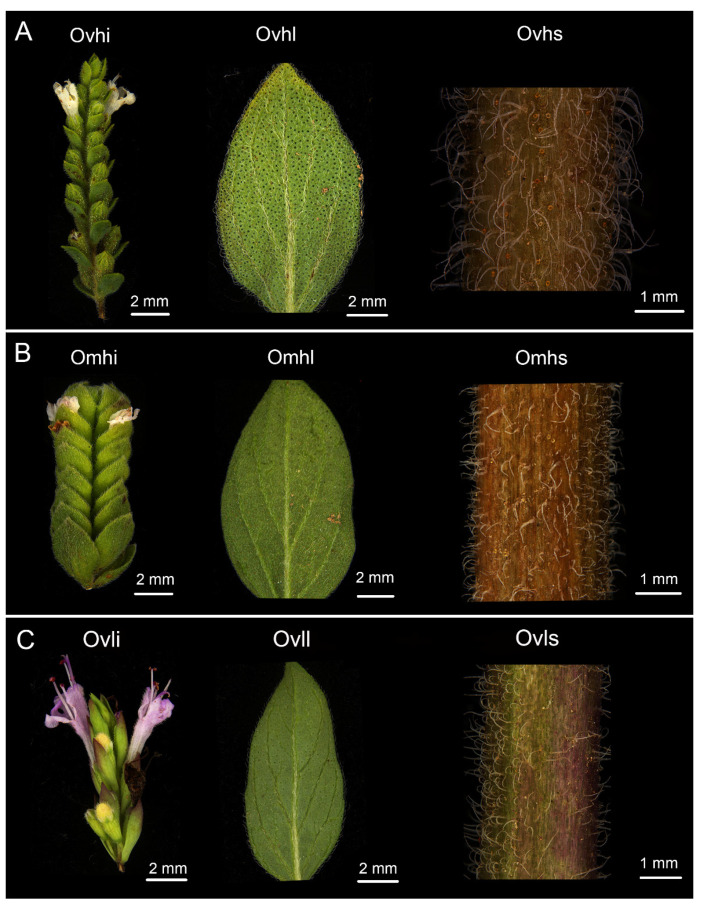
Inflorescences, leaves, and stems of three oregano plants ((**A**) Ovh; (**B**) Omh; (**C**) Ovl).

Oregano plants had white or purple flowers, an ovate shape, dark green leaves, and pubescent stems (Figure 1). In the Lamiaceae family, glandular trichomes principally produce EOs. Consequently, one of the obvious morphological characteristics of *Origanum* plants was the presence of non-glandular and glandular trichomes covering the aerial parts (Figure 1). Large quantities of specialized metabolites (i.e., EOs) are synthesized, stored, and secreted in glandular trichomes [8]. The density and size of glandular trichomes are important factors that regulate the production of EOs. Traditionally, EOs are obtained from plant glandular trichomes by steam distillation, and the number of glandular trichomes in the organs of oregano is linearly associated with EO yield. Therefore, greater the number of glandular trichomes in the organs, higher the amount of EOs derived from the organs by distillation. Our observations showed that glandular trichomes were abundant on the inflorescence (i.e., sepal, petal, and bract) and leaves, whereas their density was reduced on the stems (Figure 1). This may be explained by molecular regulatory mechanisms involving genetic variants related to the development of glandular hair and terpene synthase genes [14].

Interestingly, in this study, we observed differences in the density of glandular trichomes on the leaves of the three oregano plants (Figure 1). Overall, the density of glandular trichomes decreased in the following order: Ovhl > Omhl > Ovll. The number of glandular trichomes on the leaves of aromatic plants was linearly associated with their EO yields. As a result, the greater the number of glandular trichomes on the leaves, the higher the amount of EOs derived from the leaves by distillation [15]. Importantly, the density of glandular trichomes on the leaf surface is functionally associated with transpiration, leaf overheating, insect attack, and UV-B radiation, among other factors. The shape, density, size, and position of the glandular trichomes, as well as the oily properties, chemical constitutions, and fragrance characteristics of the EOs are carefully controlled parameters in aromatic plants, and further studies are needed to improve our understanding of the structural, functional, and ecological features of glandular trichomes and their secretions. Additionally, genetic engineering could be used to increase glandular trichome density and enhance EO yields without any adverse effects on plant growth [14].

### 3.2. Chemical Composition of OEOs

In this study, monoterpenes and sesquiterpenes were the main OEO compounds (Table 1). In particularly, in Ovh, the monoterpene carvacrol was ubiquitous and found in the inflorescences, leaves, and stems, accounting for 90.28%, 86.03%, and 79.31% of the total EOs, respectively, followed by its biosynthetic precursor p-cymene, which accounted for 2.16%, 3.37%, and 5.65% of the total EOs, respectively. A previous study reported that carvacrol accounted for 30.73% of total EOs in the OEOs of leaves and flowers, but only 6.02% of total EOs in the stem [16]. Among Omh EOs, terpinen-4-ol contents were high in the inflorescences (25.05%), leaves (22.13%), and stems (21.42%), followed by thymol contents in the inflorescences (18.74%), leaves (14.31%), stems (18.72%). Among the EOs of Ovl, sesquiterpenes were the dominant constituents in the inflorescences and leaves, including β-caryophyllene, which accounted for 13.99% and 10.51% of the total EOs, respectively; germacrene D, which accounted for 11.13% and 11.26% of the total EOs, respectively; and elixene, which accounted for 25.27% and 13.69% of the total EOs, respectively. Additionally, Ovl EOs contained some monoterpene compounds, including γ-terpinene (2.62–12.28%) and terpinen-4-ol (2.17–7.21%). EO yield of Ovls was extremely low (<0.1%). We cannot obtain enough EOs of Ovls using a Clevenger apparatus. Overall, we observed larger variations in the chemical profiles of OEOs among different cultivars (genotypes) than different parts, as expected based on the similar growth conditions used for the plants. Individual plants rich in EOs typically accumulate large amounts of phenolic monoterpenes derived from the ‘cymyl’-pathway (mainly carvacrol or thymol and their precursors γ-terpinene and p-cymene, respectively) [17]. This is consistent with our experimental results; Ovh and Omh had high EO yields and contained many phenolic compounds (i.e., carvacrol or thymol). Such high-quality plant materials have wide commercial applications in the food, cosmetics, and pharmaceutical fields. Indeed, the economic yield of EO-bearing plants depends on four key factors: the accumulation of dry material, ratio of economically valuable parts, EO amount, and relative contents of valuable compounds [18].

Plants produce large quantities of metabolites in an environment-dependent and spatiotemporally dependent manner [19]. The concentrations and constituents of EO compounds usually vary due to many factors, such as species, harvest season, geographical location, soil conditions, and climatic and growth conditions [20]. Higher carvacrol contents are associated with better EO yields. Moreover, oregano tends to have increased oil contents at higher temperatures and light intensities and with longer daytime growth. The yield of EOs is higher during the flowering period than during the vegetative period. The crop yields of medicinal and aromatic plants and the quantities and qualities of EO active substances can also be affected by biofertilization [21]. Nitrogen fertilization decreases carvacrol content but increases thymol content [22]. Nitrogen fertilization also decreases the bioaccessibility of phenolic compounds. As a result, increased nitrogen content leads to a decrease in the EO content [23]. Therefore, it is important to increase EO output through reasonable cultivation measures.

In this study, we found that Ovh and Omh were rich in bicyclic monoterpene cis-sabinene hydrate derived from the biosynthetic “sabinyl” pathway, whereas the phenolic monoterpene carvacrol, arising from the “cymyl” pathway, was a distinctive feature of oregano. The phenolic monoterpene alcohol thymol is derived from α-terpinene, the product of a single monoterpene synthase [24,25]. The enormous chemical polymorphism of *Origanum* offers an extensive selection of compounds for the production of specific monoterpenes as fine chemicals and new odor and flavor profiles.

**Figure 2:**
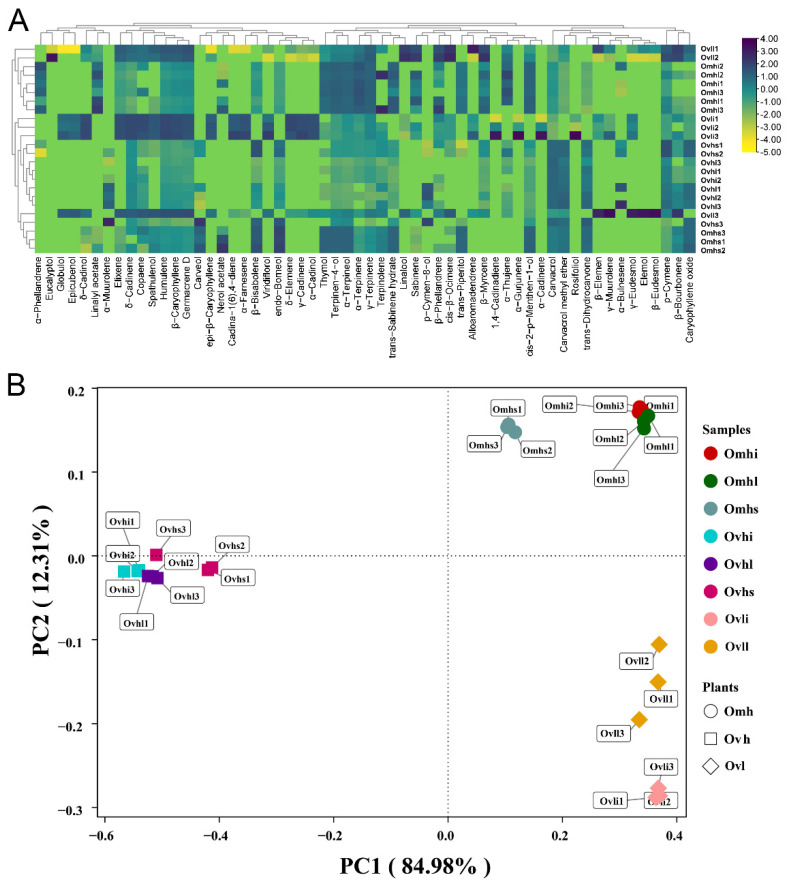
Heatmap (**A**) and principal component analysis (PCA) map (**B**) based on the chemical components of oregano essential oils (OEOs).

A PCA model (Figure 2B) was constructed from 24 samples across different cultivars and parts. The 24 samples were projected as colored nodes, and the different cultivars could be clearly classified, suggesting that the EO chemical profiles of the three cultivars were quite different. However, there were also minor differences among various parts of the same oregano cultivar. The stems (Ovhs, Omhs) were quite different from the other parts. Additionally, Ovli and Ovll were clearly distinct relative to other parts of the same cultivar.

### 3.3. Characterization of Common and Unique OEO Components

**Figure 3:**
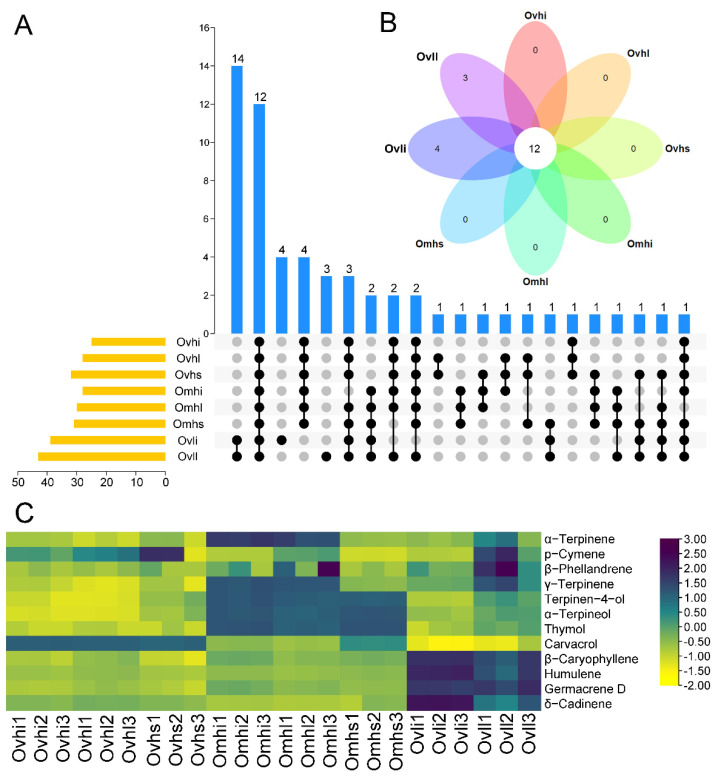
UpSet plot (**A**) and flower plot (**B**) based on amounts of chemical components in OEOs. Heatmap (**C**) of shared component contents in all samples.

UpSet analysis was then performed to visualize the distribution of common and unique components of different parts from the three oregano cultivars (Figure 3A). The results showed that the number of components present in each OEO ranged from 28 to 43. Of these, the majority of components were common in more than one sample, such as trans-sabinene hydrate, endo-borneol, trans-dihydrocarvone, and β-bisabolene, which were present in different parts of all Ovh and Omh samples, but not in Ovl. Twelve components, including α-terpinene, p-cymene, β-phellandrene, γ-terpinene, terpinen-4-ol, α-terpineol, thymol, carvacrol, β-caryophyllene, humulene, germacrene D, and δ-cadinene, were shared by the inflorescences, stems, and leaves from Ovh, Omh, and Ovl. Additionally, components such as linalool, β-elemen, and α-farnesene were common to Ovli and Ovll. There were four common components (i.e., trans-Sabinene hydrate, endo-Borneol, trans-Dihydrocarvone, β-Bisabolene) among the different parts of Ovh and Omh. Thus, we concluded that the similarity of the compounds between Ovh and Omh was higher than with Ovl. Certain components, e.g., α-cadinene (Ovli) and elemol (Ovll), were unique to specific samples (Figure 3B). The chemical profiles of the 12 shared EO components from all samples are presented in Figure 3C. Ovhi, Ovhl, and Ovhs had the highest carvacrol contents, whereas Omhi, Omhl, and Omhs showed the highest α-terpinene, γ-terpinene, terpinen-4-ol, α-terpineol, and thymol contents. Furthermore, Ovli and Ovll showed the highest sesquiterpene contents, including β-caryophyllene, humulene, germacrene D, and δ-cadinene. These results suggested that the many chemical components of EOs from different oregano cultivars and parts were similar but showed different yields.

### 3.4. Multivariate Statistical Analysis of EO Chemical Profiles

**Figure 4:**
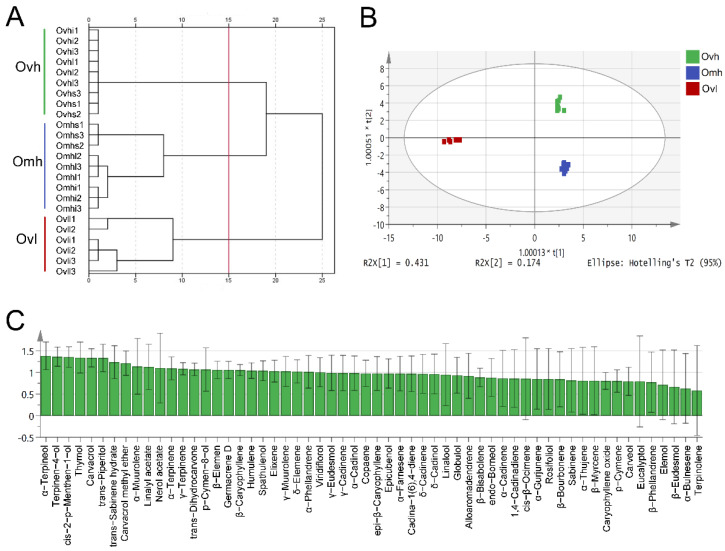
Dendrogram (**A**), score plots (**B**), and variable importance in projection (VIP) values (**C**) from orthogonal projections to latent structures discriminant analysis (OPLS-DA) based on the chemical profiles of the three OEOs.

Dendrogram analysis of all EO samples yielded three main groups (Figure 4A). The different parts of the same cultivar clustered into a large group, which we then designated Ovh, Omh, and Ovl. These results indicated that the chemical compositions of EOs from different parts were relatively similar, particularly when compared with other oregano cultivars. A supervised OPLS-DA statistical method was then used to identify components among OEOs of different cultivars (Figure 4B).

The variable importance in projection (VIP) value is a parameter for screening chemical markers and is used to determine the contributions of chemical components in multivariate statistical analyses [26]. Figure 4C shows the VIP values of each OEO component; vital components were identified based on higher VIP values (≥1). Among these, α-terpineol (1.38458), terpinen-4-ol (1.36273), cis-2-p-menthen-1-ol (1.35627), thymol (1.34232), and carvacrol (1.33647) showed significant contributions to the classification via the OPLS-DA model. Importantly, these compounds may be appropriate chemical markers for distinguishing the EOs of different oregano cultivars. The chemical markers from the aerial parts and roots of EOs from fennel and dill have been identified previously using the OPLS-DA model [13]. Overall, this comprehensive, multidimensional analysis of OEOs provides important insights into the screening of specific compounds and the evaluation of phytochemical characteristics in different samples.

### 3.5. Antibacterial Activity of OEOs

**Figure 5:**
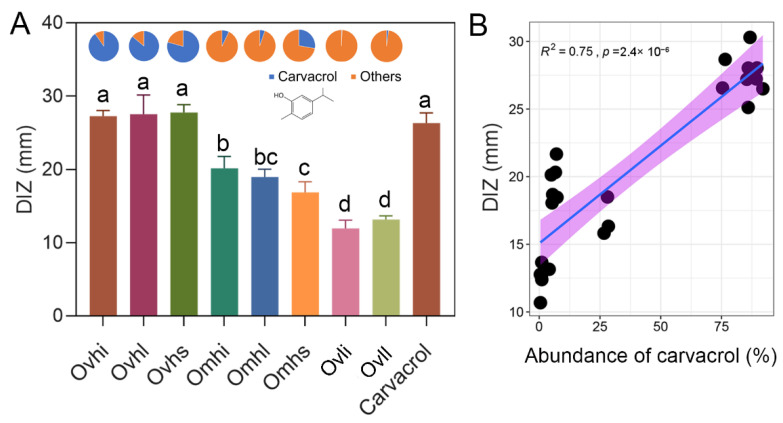
Antibacterial activities of OEOs. Diameter of the inhibitory zone (DIZ) statistical data (**A**) for OEOs against *S. aureus* and relationships of carvacrol and DIZ (**B**). Discs measured 6 mm in diameter, and values represent means ± standard deviation (*p* < 0.05).

The antibacterial activities of different OEOs and carvacrol against *S. aureus* were determined by measuring DIZ and MIC (Figure 5; Table 2). The results showed that *S. aureus* exhibited different levels of susceptibility to EOs and standards, with halos ranging from 6.96 to 27.75 mm (Figure 5A). Overall, as a gram-positive bacterium, *S. aureus* was susceptible to most OEOs and the main chemical components. While the different parts of the same oregano cultivar showed relatively similar antibacterial activity, striking differences were observed between the three oregano cultivars, with Ovh showing the highest activity against *S. aureus* (Table 2). EOs rich in cymyl compounds, mainly carvacrol, thymol, and terpene-4-ol, had stronger antibacterial capacities than sesquiterpene-rich OEOs. Within the same plant, owing to the different proportions of the main components, there were also differences in antimicrobial properties of the different parts (e.g., Omhi and Omhs).

Differences in antibacterial activity may be caused by variations in the chemical compositions of the OEOs used in this study. By comparing the relationships between carvacrol contents and antibacterial activities in OEOs of different cultivars and parts, we found that carvacrol had a significant positive correlation with antibacterial activity (by DIZ; Figure 5B). Previous studies have suggested that the active components of EOs may bind to the cell surface and then penetrate to target sites, potentially the plasma membrane and membrane-bound enzymes, resulting in disruption of the cell wall structure [27]. In the current study, we found that OEOs containing high levels of carvacrol (e.g., Ovhi, Ovhl, and Ovhs) had essentially the same antibacterial effects as the single carvacrol standard. This result suggested that natural OEOs with high oil yields and high antibacterial activities may have broad application prospects.

The main component, carvacrol, was found to have surprisingly high antibacterial activity. Its antibacterial mechanism involves disruption of the bacterial membrane and leakage of intracellular contents, resulting in death, and the compound is generally considered safe for consumption; thus, carvacrol has been approved as a food flavoring agent and has been applied a bactericide in food and feed [28]. Interestingly, in previous studies, OEO and carvacrol were also found to have significant inhibitory effects on drug-resistant bacteria [29,30]. Given the heterogeneous composition of OEOs and the antimicrobial activities of many OEO components, it seems unlikely that there is only one mechanism of action or that only one component is responsible for the antimicrobial action. At the same time, the various chemical components contained in plant EOs often have synergistic effects, and the diversity of antibacterial targets can greatly reduce the resistance of bacteria [31,32].

Further work is required to fully understand the mechanisms involved in order to justify the real applications of EOs in food practice as natural antibacterial agents [33]. EOs have promising applications as potential antibiotic alternatives. In further studies, in-depth validation and interpretation of the molecular mechanisms of EOs against bacteria is necessary. Coating technology with EOs should also be explored for application in the food industry.

## 4. Conclusions

In summary, our results indicated that the different oregano samples can be quickly and accurately distinguished by chemometrics. Through a multidimensional exploration, we found that differences in the chemical compositions of the OEOs of different parts within the same cultivar were smaller than those between different cultivars. In addition, different OEOs exhibited variations in antibacterial activities, which may be closely related to their carvacrol content. This result implied that carvacrol played an important role in the biological activities of OEOs. Among the samples in this study, Ovh, especially its inflorescences and leaves, was the preferred material for EO production due to high EO yields and carvacrol contents. Omh can be used as flavoring agent. Ovl was not suitable for extracting EOs, but it may be a good ornamental cultivar. Analyzing the EO yields of different plants and plant parts as well as differences in the chemical compositions and biological activities of the EO components could effectively guide the application of EOs as food additives.

## Figures and Tables

**Table 1:** Chemical compositions of essential oils derived from the inflorescences, leaves, and stems of three oregano cultivars.

Compounds	RI	Ovhi	Ovhl	Ovhs	Omhi	Omhl	Omhs	Ovli	Ovll
α-Thujene	932	0.06 ± 0.06	0.08 ± 0.03	0.19 ± 0.26	1.01 ± 0.4	0.97 ± 0.97	-	-	0.33 ± 0.23
Sabinene	973	-	-	-	0.29 ± 0.26	0.88 ± 0.6	0.05 ± 0.01	0.15 ± 0.14	1.65 ± 1.35
β-Myrcene	991	0.08 ± 0.01	0.09 ± 0.04	0.13 ± 0.11	0.24 ± 0.04	0.48 ± 0.04	-	-	0.87 ± 0.65
α-Phellandrene	1006	-	-	0.07 ± 0.08	0.49 ± 0.1	0.37 ± 0.15	-	-	-
α-Terpinene	1018	0.39 ± 0.05	0.07 ± 0.13	0.5 ± 0.43	11.27 ± 0.78	8.74 ± 1.54	0.6 ± 0.07	0.68 ± 0.13	2.92 ± 1.99
p-Cymene	1026	2.16 ± 0.3	3.37 ± 0.51	5.65 ± 4.58	0.79 ± 0.01	2.02 ± 0.15	0.51 ± 0.02	0.78 ± 0.04	5.72 ± 3.54
β-Phellandrene	1030	0.04 ± 0.06	0.06 ± 0.06	0.1 ± 0.17	0.25 ± 0.25	1.3 ± 1.26	0.04 ± 0.07	0.3 ± 0.15	1.67 ± 1.08
Eucalyptol		-	-	-	-	-	-	-	0.18 ± 0.16
cis-β-Ocimene	1038	-	-	0.04 ± 0.04	-	-	0.04 ± 0.04	0.14 ± 0	3.47 ± 2.16
γ-Terpinene	1060	0.77 ± 0.2	0.23 ± 0.06	0.79 ± 0.56	13.79 ± 0.99	13.3 ± 0.16	1.69 ± 0.11	2.62 ± 0.12	12.28 ± 6.59
trans-Sabinene hydrate	1069	0.3 ± 0.12	0.4 ± 0.08	1.02 ± 0.1	6.54 ± 3.76	14.6 ± 0.5	9.36 ± 0.14	-	-
Terpinolene	1091	0.12 ± 0.03	0.26 ± 0.15	0.19 ± 0.17	-	1.8 ± 1.57	0.41 ± 0.02	0.15 ± 0.13	0.23 ± 0.21
Linalool		-	-	-	-	-	-	0.21 ± 0.04	0.98 ± 0.28
cis-2-p-Menthen-1-ol	1124	0.09 ± 0.03	0.08 ± 0.02	0.23 ± 0.07	2.28 ± 0.16	2.58 ± 0.18	2.46 ± 0.12	-	0.27 ± 0.22
endo-Borneol	1170	0.17 ± 0.01	0.25 ± 0.02	0.53 ± 0.12	0.18 ± 0	0.19 ± 0.01	0.88 ± 0.04	-	-
Terpinen-4-ol	1181	1.36 ± 0.32	1.08 ± 0.11	3.64 ± 0.91	25.05 ± 1.55	22.13 ± 0.98	21.42 ± 0.76	2.17 ± 0.1	7.21 ± 1.32
p-Cymen-8-ol	1188	-	0.17 ± 0.01	0.14 ± 0.04	-	-	-	-	-
α-Terpineol	1194	0.19 ± 0.04	0.17 ± 0.02	0.78 ± 0.24	4.71 ± 0.08	4.15 ± 0.14	4.77 ± 0.08	0.75 ± 0.09	1.58 ± 0.21
trans-Dihydrocarvone	1201	0.24 ± 0.05	0.31 ± 0.04	0.33 ± 0.06	0.1 ± 0	0.09 ± 0.02	0.32 ± 0.01	-	-
trans-Piperitol	1211	-	-	0.12 ± 0.06	0.72 ± 0.04	0.78 ± 0.04	0.97 ± 0.01	-	-
Carveol	1223	-	0.06 ± 0.02	0.12 ± 0.05	-	-	0.13 ± 0.01	-	-
Carvacrol methyl ether	1248	0.63 ± 0.1	0.99 ± 0.18	0.87 ± 0.07	0.05 ± 0	0.05 ± 0.01	0.6 ± 0.03	-	0.17 ± 0.03
Linalyl acetate	1259	-	-	-	0.5 ± 0.18	1.14 ± 0.14	0.2 ± 0.09	-	0.17 ± 0.15
Thymol	1294	0.1 ± 0.17	0.23 ± 0.2	0.21 ± 0.37	18.74 ± 0.62	14.31 ± 0.75	18.72 ± 0.08	0.45 ± 0.4	2.49 ± 0.51
Carvacrol	1304	90.28 ± 1.5	86.03 ± 0.66	79.31 ± 5.83	7.01 ± 0.32	5.25 ± 0.28	27.73 ± 0.94	0.67 ± 0.33	2 ± 1.81
δ-EIemene		-	-	-	-	-	-	0.83 ± 0.04	0.43 ± 0.11
Nerol acetate	1368	-	-	-	0.09 ± 0.1	0.15 ± 0.09	0.26 ± 0.13	-	-
Copaene	1382	0.05 ± 0.04	0.07 ± 0.01	0.05 ± 0.01	0.11 ± 0.1	-	0.12 ± 0.1	0.63 ± 0.06	0.33 ± 0.13
β-Bourbonene	1393	0.03 ± 0.02	0.15 ± 0.02	0.05 ± 0.04	-	0.05 ± 0.01	0.15 ± 0.02	0.1 ± 0.08	0.85 ± 0.19
β-Elemen		-	-	-	-	-	-	0.44 ± 0.01	0.48 ± 0.1
α-Gurjunene		-	-	-	-	-	-	0.18 ± 0.04	-
β-Caryophyllene	1428	1.36 ± 0.17	1.95 ± 0.18	0.93 ± 0.23	2.69 ± 0.05	2.03 ± 0.13	2.42 ± 0.1	13.99 ± 0.18	10.51 ± 2.41
Alloaromadendrene		-	-	-	-	-	-	0.3 ± 0.09	0.35 ± 0.28
Humulene	1462	0.16 ± 0.02	0.23 ± 0.02	0.11 ± 0.02	0.24 ± 0	0.19 ± 0.02	0.26 ± 0.02	3 ± 0.09	1.95 ± 0.53
epi-β-Caryophyllene		-	-	-	-	-	-	1.48 ± 0.08	0.55 ± 0.46
Cadina-1(6),4-diene		-	-	-	-	-	-	0.33 ± 0.03	0.09 ± 0.09
Germacrene D	1484	0.41 ± 0.03	0.66 ± 0.07	0.32 ± 0.17	1.34 ± 0.11	0.92 ± 0.09	0.76 ± 0.03	11.13 ± 0.65	11.26 ± 2.56
γ-Muurolene		-	-	-	-	-	-	0.85 ± 0.17	1.01 ± 0.41
α-Bulnesene	1502	-	0.11 ± 0.1	0.06 ± 0.05	0.06 ± 0.04	-	-	-	-
Elixene	1505	-	-	-	0.79 ± 0.04	0.56 ± 0.03	0.34 ± 0.12	25.27 ± 0.93	13.69 ± 2.67
α-Muurolene	1507	0.04 ± 0.04	0.1 ± 0	0.07 ± 0.07	-	-	-	-	-
α-Farnesene		-	-	-	-	-	-	3.39 ± 0.13	0.98 ± 0.91
β-Bisabolene	1514	0.19 ± 0.01	0.34 ± 0.02	0.72 ± 0.08	0.32 ± 0.01	0.29 ± 0.03	0.72 ± 0.05	-	-
γ-Cadinene		-	-	-	-	-	-	2.33 ± 0.1	0.77 ± 0.29
δ-Cadinene	1530	0.38 ± 0.04	0.51 ± 0.02	0.36 ± 0.06	0.11 ± 0.01	0.05 ± 0.01	0.24 ± 0.21	7.75 ± 0.11	2.35 ± 0.88
1,4-Cadinadiene		-	-	-	-	-	-	0.21 ± 0.02	-
α-Cadinene		-	-	-	-	-	-	0.45 ± 0.01	-
Elemol		-	-	-	-	-	-	-	0.43 ± 0.28
Globulol		-	-	-	-	-	-	1.88 ± 0.36	0.74 ± 1.19
Spathulenol	1586	-	-	0.08 ± 0	-	0.06 ± 0.05	0.73 ± 0.19	4.6 ± 0.28	3.47 ± 1.62
Caryophyllene oxide	1592	0.17 ± 0.04	0.91 ± 0.11	1.22 ± 0.12	-	0.09 ± 0.01	1.15 ± 0.21	0.16 ± 0.01	0.64 ± 0.61
Viridiflorol		-	-	-	-	-	-	1.05 ± 0.22	0.51 ± 0.3
Rosifoliol		-	-	-	-	-	-	0.33 ± 0.08	-
Epicubenol		-	-	-	-	-	-	0.25 ± 0.01	0.12 ± 0.14
γ-Eudesmol		-	-	-	-	-	-	0.3 ± 0.02	0.29 ± 0.21
δ-Cadinol	1648	-	-	-	-	-	0.2 ± 0.12	4.25 ± 0.16	1.09 ± 0.61
β-Eudesmol		-	-	-	-	-	-	-	0.4 ± 0.32
α-Cadinol		-	-	-	-	-	-	4.43 ± 0.01	1.42 ± 0.86
Total		99.77 ± 0.13	98.98 ± 0.24	98.93 ± 0.31	99.75 ± 0.16	99.54 ± 0.55	98.25 ± 0.54	98.96 ± 0.32	98.95 ± 0.7
EO yields	5.83 ± 0.15	2.23 ± 0.06	0.2 ± 0	2.3 ± 0.1	1.03 ± 0.12	0.1 ± 0	0.27 ± 0.06	0.1 ± 0	

Notes: ‘-’: not detected; data are means ± standard deviations; n = 3. Compound identification was based on the NIST 17 mass spectral database and the retention index (RI).

**Table 2:** MICs of OEOs against *S. aureus*.

Bacteria	Concentrations of OEO (mg/mL)											
	0.0625	0.125	0.25	0.5	1	2	4	8	16	32	64	128
Ovhi	+	-	-	-	-	-	-	-	-	-	-	-
Ovhl	+	-	-	-	-	-	-	-	-	-	-	-
Ovhs	+	+	-	-	-	-	-	-	-	-	-	-
Omhi	+	+	+	+	+	-	-	-	-	-	-	-
Omhl	+	+	+	+	+	-	-	-	-	-	-	-
Ovli	+	+	+	+	+	+	+	+	-	-	-	-
Ovll	+	+	+	+	+	+	+	-	-	-	-	-

Note: “+”: observed bacterial growth, “-”: no visible bacterial growth. Sufficient Omhs was not obtained through EO extraction for MIC test.

## Data Availability

Not applicable.
